# The quality and quantity of staff-patient interactions as recorded by staff. A registry study of nursing documentation in two inpatient mental health wards

**DOI:** 10.1186/s12888-019-2236-y

**Published:** 2019-08-14

**Authors:** Kjellaug K. Myklebust, Stål Bjørkly

**Affiliations:** 1 Faculty of Health Sciences and Social Care, Molde University College, Box 2110, 6402 Molde, Norway; 2 Centre for Forensic Psychiatry, Oslo University Hospital, Oslo, Norway

**Keywords:** Attunement, Empathy, Mental health care, Staff-patient relationship, Nursing records, Progress notes, Psychiatric care, SESPI, Therapeutic relationship

## Abstract

**Background:**

Therapeutic staff-patient interaction is fundamental in psychiatric care. It is recognized as a key to healing in and of itself, or a premise to enhance psychiatric treatment adherence. Still, little is known about how these interactions are recorded in nursing documentation. The purpose of the study was to assess the quality and quantity of staff-patient interactions as recorded in progress notes in nursing documentation.

**Methods:**

The study has an observational registry study design. A random sample of 3858 excerpts was selected from progress notes in 90 patient journals on an acute psychiatric unit and an open inpatient district psychiatric centre (DPC) in Norway. The Scale for the Evaluation of Staff-Patient Interactions in progress notes (SESPI) was used to assess the progress note excerpts. It is developed to assess the quality and quantity in excerpt descriptions of staff-patient interactions in terms of empathic attunement. Descriptive statistics were calculated for the total sample and for each ward separately. Ordinal and multinomial logistic regression were used to estimate control for shift type, staff education level, and type of hospital ward.

**Results:**

Only 7.6% of the total number of excerpts (*N* = 3858) described staff-patient interactions sufficiently to analyze them in terms of attunement. Compared to the DPC, the acute ward reported more staff-patient interactions. The evening excerpts reported more successful types of attunement than those from the night shifts. Education level did not contribute significantly to our models.

**Conclusion:**

These findings present a unique insight into the quality and quantity of mental health nursing documentation regarding staff-patient interactions. Therapeutic interactions where staff tried to attune to the patients were rarely described. However, this is the first study measuring nursing documentation with the SESPI, and more studies are required to validate the scale and our findings. One potential clinical implication of this research is the development of a scale that personnel in psychiatric wards can have for evaluation of the quality of their reporting practice with emphasis on staff-patient interactions. By regular use this may help keeping up emphasis on emphatic attunement in milieu treatment contexts.

**Electronic supplementary material:**

The online version of this article (10.1186/s12888-019-2236-y) contains supplementary material, which is available to authorized users.

## Background

Staff-patient interactions is a highly relevant topic for current research in inpatient mental health contexts [1–3]. In spite of a broad common understanding among staff and patients about the importance of therapeutic staff-patient relationships, it is unclear how, and to what extent, therapeutic staff-patient interactions unfold in clinical practice [4–6]. When it comes to the most tense and demanding situations, staff-patient interactions play a central role both as antecedents of aggressive episodes and as measures to prevent their escalation [7, 8]. Some studies have suggested positive effects of therapeutic staff-patient interactions relating to, for instance, schizophrenia or depression, but according to these studies, research measuring therapeutic staff-patient interactions are sparse [9, 10]. From a pharmacological perspective a therapeutic relationship seems important for treatment adherence [11]. A systematic review of studies conducted in a number of hospital units revealed discrepancies between nurses’ and patients’ views of good qualitative care and highlighted a need for further research in order to understand differing perceptions and to improve nurse-patient interactions [12]. Researchers have called for better operationalization of the term therapeutic engagement between nurses and patients to measure these aspects in acute mental health wards [13]. Qualified nurses constitute the highest number of persons working in mental health units, but ward staff also comprises professionals with other educational backgrounds such as, for example, social workers and assistant nurses [14, 15]. Nursing documentation is the record of care that was planned or given to individual patients by qualified nurses or other caregivers under the direction of a qualified nurse [16]. Accordingly staff members with different educational backgrounds are reporting in the nursing documentation. Ideally, these reports should contain a continuous narrative of a patient’s experience, how the staff understood the situation, and how the staff dealt with it. All instances of psychosocial support are supposed to be reported [17].

Against this background and given that the therapeutic relationship has been considered a core feature of mental health nursing and mental health care in general (e.g.[18–20]), it could be expected that staff-patient communication would be a sustained element in nursing documentation in mental health care services. Additionally, it could be anticipated that researchers would pay attention to descriptions of staff-patient communications when assessing nursing documentation.

There are few studies examining descriptions of staff-patient interactions in nursing documentation. The most relevant quantitative study evaluated the frequency of documented psychosocial interventions in acute care settings, but only 3.8% of the data was collected in mental health services [21]. This study reported a high frequency of interventions that were initiated to meet patients’ basic needs and to create a basis for a therapeutic relationship. However, the researchers considered the documented interventions insufficient when it came to dealing with patients’ emotional difficulties.

Researchers have focused on different aspects of structure, process, and content when assessing nursing documentation [22]. For example, issues that were explored included percentages of numbers of progress notes correctly signed and dated, the most frequently documented patients’ problems across different hospital contexts, and documentation of vital somatic variables on psychiatric wards [23–25]. Others paid attention to the use of standardized nursing diagnoses in inpatient psychiatric care [26–28]. However, none of these studies addressed the content of described therapeutic staff-patient interactions or thematized staff-patient relationships in nursing documentation. A thematic analysis of care plans for patients previously involved in aggressive incidents touched on the topic we were searching for. Offering a conversation with a patient was the most frequently used de-escalation strategy (17%), but these de-escalation interactions were often described in vague and general terms [29]. Qualitative studies from different mental health contexts highlighted that nursing documentation primarily comprised nurses’ observations of patients’ behaviors and provided only limited information about staff-patient interactions [30–32].

Considering the strong emphasis on therapeutic staff-patient relationships in the mental health literature, it is remarkable that therapeutic interactions have barely been investigated in quantitative studies of nursing documentation in inpatient mental health care. Nursing documentation is an important source for evaluating and developing mental health care, and for this reason, examining the quality and quantity of described staff-patient interactions is highly relevant. Progress notes in mental health services are the staff’s reports in the electronic patient records. They are usually written for every shift and, as such, they are an essential part of nursing documentation. The following is an example of an excerpt of a progress note from the current study:

*Patient A was agitated and restless. He talked to himself, burst into laughter several times, and started to kick into thin air. When the nurse told him to stop this behavior, Patient A got very angry and upset.*

This excerpt illustrates that not all interactions turn out to be therapeutic. In the current study, the staff-patient interactions in the progress notes were analyzed from an attunement perspective. Self-psychology is a psychotherapeutic approach that emphasizes the paramount importance of empathy and attunement in creating and sustaining a positive therapeutic relationship [33–35]. Attunement is described as the key to an empathic interaction. The process of attuning to someone involves recognizing the other’s affective mode and synchronizing to that person’s emotional experience. Attunement is suggested to be one of the factors that can make a positive difference in patients’ experiences of involuntary admissions and treatment [36, 37]. Furthermore, mental health staff have emphasized the importance of attuning to a patient at times when a patient’s behavior is characterized by psychotic communication [38]. They tried to listen beyond the patient’s seemingly disturbed communication and to focus on the feelings expressed through this behavior.

The aims of this study were to use the Scale for the Evaluation of Staff-Patient Interactions in Progress Notes (SESPI) to

1. Identify the extent and quality of described patient experiences.
2. Assess the extent of reported staff attunement approaches in response to the described patient experiences.
3. Monitor the quality of the reported staff-patient interactions with a primary focus on the degree of failed or successful attunement.

## Methods

### Setting and sample

This study reviewed excerpts of progress notes from the nursing documentation from a locked acute psychiatric unit and an open inpatient district psychiatric centre (DPC) in Norway. These two hospital units differed considerably regarding severity of psychotic symptoms of the patients and challenging behaviors. Further, they differed pertaining to involuntary versus voluntary admission and treatment with or without use of coercion. Due to the void in the literature concerning staff-patient interactions in nursing documentation, these wards were chosen in order to assess the sensitivity of the SESPI in different treatment contexts. The reporting from this research was based on the checklist Strengthening the Reporting of Observational Studies in Epidemiology (STROBE). There were 12 beds at the locked acute ward and 14 beds at the DPC. Nurses with or without degrees in mental health care constituted the largest group of staff. Some of the staff had bachelor degrees in other health or social sciences, with or without further mental health education; others were healthcare assistants and assistants without formal education. Altogether, there were about 50 mental health staff members at the acute ward and 40 at the DPC, all of whom were responsible for writing progress notes in the electronic patient records.

A pre-study of progress notes (*N* = 1051) retrieved from 10 electronic patient journals from each of these two wards had been previously conducted for instrument development and testing. The results from the pre-study showed significant differences between the two wards regarding documentation of staff-patient interactions in terms of categories of failed or successful attunement. The differences were measured with a 4-point scale ranked from Failed attunement (coded 1) to Successful attunement (coded 4). Mean score for the acute ward excerpts from progress notes was 2.77. This is a score between Partially failed attunement (coded 2) and Partially successful attunement (coded 3). As for the DPC mean score was 3.05, which is very close to Partially successful attunement, (t (76) = 2.091, *p* = .040). This means that the excerpts from the DPC described more often successful attunement compare to the excerpts from the acute ward. In the current study, we wanted to randomize a number of excerpts from progress notes. The difference between the two wards that were found in the pre-study, constituted the basis for estimating sample size of excerpts for each ward. To estimate the sample size needed, separate statistical strength calculations were conducted for each of the two wards based on data from the pre-study. A sample of 130 excerpts per ward that contained complete descriptions of staff-patient interactions, the final step in SESPI (Step 4), was required to obtain an 80% likelihood that the study would produce a statistically significant effect. The excerpts analyzed in the current study were randomized from 30 electronic patient journals from the acute psychiatric ward and 60 journals from the DPC. The estimated number of journals needed was based on the assumption that the journals for the current study contained the same average amount of excerpts that described attunement as the journals in the pre-study. A large number of excerpts (*n* = 2974) were retrieved from the 30 electronic patient journals on the acute ward and the 60 journals from the DPC (*n* = 2823 excerpts). By using a random number generator, excerpts were randomly selected, and scored in SESPI, until we reached saturation according to the sample size calculations based on the pre-study. In order to get the required 130 excerpts that fully described staff-patient interactions in terms of attunement, we had to randomize 1929 excerpts from the pool of excerpts from the DPC (*n* = 2823). Thereafter the same number of excerpts (*n* = 1929) were randomized from the acute ward (*n* = 2974).

### Procedure for preparing progress notes for analysis and for scoring of excerpts

We used only progress notes written before staff were informed about this research (November 1, 2015). This was done to avoid research-triggered bias in the progress notes. A nurse from each ward picked the 30 or 60 journals that covered the most recent admission period of at least 14-days stay before the first of November 2015. The nurses retrieved progress notes covering the period from admittance until discharge, with an upper limit of 4 weeks. Only one admission was analyzed from each journal. The progress notes from the 30 journals at the acute psychiatric ward were written from May 3, 2015, to November 1, 2015, whereas the period for the DPC was from September 1, 2014, to November 1, 2015. According to the conditions for the study’s ethical approval all personal identifying information retrieved from the electronic patient journals was removed before copies of the anonymous progress notes were handed over to the researchers. Thus, the data used for this research did not contain any information about patients’ names, diagnoses, sex, occupations, or other sociodemographic variables. In addition, staff signatures were removed from the progress notes and only the occupation of the individual signing the note was accessible to the researchers. In line with the research aims, we wanted to exclude progress notes in which the staff and patient obviously had no opportunity to interact. The first author prepared the progress notes for analysis using the following procedure:

- Descriptions of all types of communication between staff and others were removed if the patient had not been present in the reported situation.
- Notes from conversations between patients and others than ward staff (e.g., psychiatrists, family, and friends) were removed.
- If a note contained more than one episode and different patient experiences or feelings were reported in relation to these episodes, the note was split and counted as two separate progress notes (or equal to the number of the described episodes).

After the removal procedure, the remaining content of the progress notes was included as “an excerpt”. All together there were 5797 excerpts from the two wards in the total sample for randomization. The SESPI was used to measure the quality and quantity of described experiences and staff-patient interactions in the randomized excerpts (*n* = 3858). The Cronbach’s alpha was very high for the entire instrument (.977) and the ICC was .770 when the SESPI was reliability-tested in a previous study [39].

**Fig. 1:**
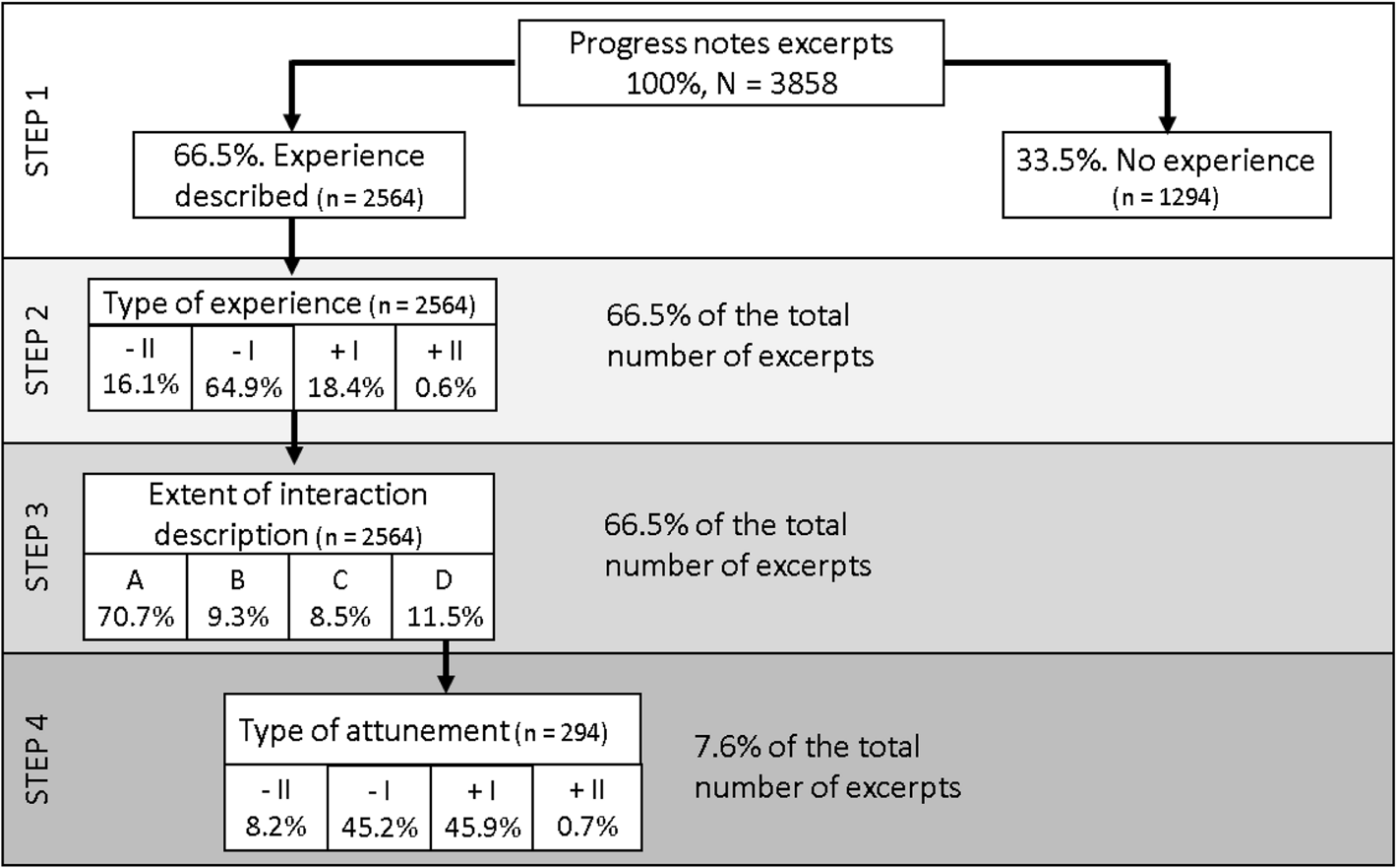
Distribution of progress notes excerpts over, and within, the four steps in the SESPI

The SESPI consists of four steps (see Fig. 1). The following excerpt, one of the 3858 that was scored in the current study, will illustrate how the excerpts were scored in the SESPI:

*Patient B said she would never recover from her mental disease and wished to die. One of the nurses promised to see how she was doing every half hour and offered to sit down and do some hobby activities together with her. After a while, Patient B started to draw a picture and explained that she had a mixture of feelings difficult to classify and describe. One part of her wanted to live, but the other part wanted to die. The nurse asked whether drawing and other activities helped her to get a distance from her thoughts. She confirmed that it was good for her to do some activities and that she felt more comfortable when she knew the nurse would come to see her and cared about how she was doing.*

Step 1 has a dichotomous scale where the excerpts were assessed in terms of whether they contained some kind of description of a patient’s experience or not. The episode with Patient B was scored in the category “experience described”.

Step 2 has a four-point scale for patient experiences ranging from - II to + II:

- II = Very uncomfortable
- I = Uncomfortable
- I = Positive
- II = Very positive

Each category is operationalized with examples in the user manual of the SESPI (See Additional file 1). The description of Patient B’s experience was given a - II because “Patient B said she would never recover from her mental disease and wished to die,” indicating a very uncomfortable experience.

In Step 3, descriptions in the excerpts of staff-patient interactions were scored in one of the following four categories:

A. No description of the staff’s approach.
B. The staff’s approach is described, but the patient’s response is not.
C. The staff’s approach is described, and the patient appears to experience it as fair enough. However, the approach is mostly oriented towards practical solutions and fails to capture the patient’s experience or feelings.
D. Both staff’s approach and patient’s response are described.

The “Patient B excerpt” was scored as category D. Only excerpts scored as category D continued on to Step 4. In Step 4, the described staff-patient interactions were assessed by four alternative categories of attunement:

- II = Failed attunement
- I = Partially failed attunement
- I = Partially successful attunement
- II = Successful attunement.

Excerpts where the staff member is described as being in an incongruent expert position and the patient’s response is strongly negative is scored – II. For instance, the excerpt with Patient A, presented in the introduction of this paper, belongs to – II category (“...a nurse told him to stop this behavior, Patient A got very angry and upset”). Category - I includes a continuum from slightly authoritative approaches to approaches where staff tried to grasp the patient’s experience, but the patient’s reaction was negative. The + I and + II categories include approaches intended to explore or confirm the patient’s experience together with positive feedback from the patient. The Patient B excerpt was given a + II because the description indicated that the nurse succeeded in meeting Patient B’s emotional needs and that the nurse’s approach opened a dialog where Patient B could reveal more of her feelings.

A nurse familiar with writing progress notes but who was not a part of the research team and the first author independently scored a sample of 90 excerpts using the SESPI. The excerpts were randomly selected from the pool of 3858. The Intraclass correlation coefficient (ICC) was applied to calculate the inter-rater reliability of the distribution of scores for the 90 excerpts across the four steps of coding. The One-way model was used. ICC between the two raters was acceptable, ICC = .76 (95% CI-0.216-1.000). Thereafter, the first author scored all of the 3858 excerpts, but consulted the second author for consensus decision throughout the scoring process.

### Data analysis/ statistics

Statistical power calculations were done with Sample Power Version 3, the ordinal analyses with STATA Version 15, and all other statistical analyses were conducted with SPSS Version 25. Descriptive statistics for all variables were calculated for the total sample (*N* = 3858), and for each of the two wards separately. Independent two-sample t-tests were used to estimate differences between continuous variables and the Pearson chi-square test, for categorical variables.

The following three independent variables were used in all the regression analysis: Hospital ward (acute vs. DPC), Shift type (day, evening, night), staffs’ educational level (bachelor degree with further education, bachelor degree, others such as healthcare assistants or assistants with no education). Binary logistic regression was used for calculating odds ratios for the independent variable in Step 1 of SESPI (did the excerpt describe the patient’s experience? yes or no). The dependent variables in Steps 2 and 4 (− II, − I, + I, and + II) were scored (separately) from 1 to 4 in STATA. They were both scored on ordinal level score scales, and the normal distribution tests were significant. Hence, ordinal regression was applied for Step 2 and 4. In Step 3, the dependent variable (A, B, C, D) was scored from 1 to 4 in SPSS with category D (both staff’s approach and patient’s response are described), as reference category. Multinomial regression was used to calculate likelihood ratios for the categories of interaction (Step 3). As shown in Fig. 1, 2564 excerpts proceeded to Step 2; thus *n* = 2564 is the total sum for the calculations of Steps 2 and 3. For Step 4, *n* = 294, see Fig. 1.

## Results

A total of 3858 excerpts from progress notes were analyzed. Figure 1 provides an overview of the distribution of excerpts across the four steps and within each step. The most noteworthy result was that in only 7.6% of the excerpts were staff-patient interactions sufficiently described to be analyzed in terms of attunement (see Fig. 1). A description of the patient’s experience was present in 66.5%, with approximately one third of the excerpts failing to cover any patient-related experiences or feelings. Only 11.5% of the excerpts that contained a description of a patient’s experience described whether the staff succeeded or failed to attune to the patient in the particular episode.

### Descriptions of patient experiences

Table 1 provides an overview of descriptive statistics for the randomized excerpts for the acute ward versus the DPC. Regarding educational level of staff that wrote the excerpts, DPC had the largest proportion of reporters with the highest level of education (41.6% with a bachelor’s degree and further education in mental health versus 17% for the acute ward, χ2 (1) = 282.52, *p* < .001). The two wards differed considerably in the number of excerpts reported from night shifts (acute with 29% vs. DPC with 8.2%; χ2 (1) = 275.46, p < .001).

**Table 1:** Descriptive statistics for the acute ward excerpts versus DPC excerpts

	Acute	DPC	Total
	n (%)	n (%)	n (%)
Independent variable: Education level staff			
Bachelor with further education	327 (17.0)	802 (41.6)	1129 (29,3)
Bachelor	869 (45.0)	856 (44.4)	1725 (44,7)
Other	733 (38.0)	271 (14.0)	1004 (26,0)
Total	1929 (100)	1929 (100)	3858 (100)
Independent variable: Shift type			
Day	619 (32.1)	915 (47.4)	1534 (39.8)
Evening	751 (38.9)	856 (44.4)	1607 (41.7)
Night	559 (29.0)	158 (8.2)	717 (18.6)
Total	1929 (100)	1929 (100)	3858 (100)
Dependent variable: Experience described			
No	688 (35.7)	606 (31.4)	1294 (33.5)
Yes	1241 (64.3)	1323 (68.6)	2564 (66.5)
Total	1929 (100)	1929 (100)	3858 (100)
Dependent variable: Type of described experience			
- II Very uncomfortable	252 (20.3)	160 (12.1)	412 (16.1)
- I Uncomfortable	829 (66.8)	834 (63.0)	1663 (64.9)
+ I Positive	155 (12.5)	318 (24.0)	473 (18.4)
+ II Very positive	5 (0.4)	11 (0.8)	16 (0.6)
Total	1241 (100)	1323 (100)	2564 (100)
Dependent variable: Type of described staff-patient interactions			
A. No staff approach	869 (70.0)	945 (71.4)	1814 (70.7)
B. Staff approach, but not the patient’s response	106 (8.5)	132 (10.0)	238 (9.3)
C. “Superficial” staff approach	104 (8.4)	114 (8.6)	218 (8.5)
D. Fully described interaction	162 (13.1)	132 (10.0)	294 (11.5)
Total	1241 (100)	1323 (100)	2564 (100)
Dependent variable: Type of described attunement			
- II Failed attunement	21 (13.0)	3 (2.3)	24 (8.2)
- I Partially failed attunement	79 (48.8)	54 (40.9)	133 (45.2)
+ I Partially successful attunement	61 (37.7)	74 (56.1)	135 (45.9)
+ II Successful attunement	1 (0.6)	1 (0.8)	2 (0.7)
Total	162 (100)	132 (100)	294 (100)

There was a significant difference between the wards concerning the number of excerpts that described experiences (acute = 64.3% vs. DPC = 68.6%; χ2 (1) = 7.82, *p* = .005). However, when controlling for educational level and shift type, the DPC excerpts had about 18% decreased odds for reporting experiences compared to the acute ward (OR = .822, *p* = .017, 95% CI = .700–.966; see Table 2). Excerpts reported in day and evening shifts had increased odds for reporting experiences compared to the excerpts reported in night shifts (day shift, OR = 5.308, *p* < .001, 95% CI = 4.307–6.543; evening shift: OR = 6.474, p < .001, 95% CI = 5.260–7.969). The Hosmer and Lemeshow test was significant for this logistic regression model, but, as with any statistical test, the power increases with sample size. The size of the current data set (*N* = 3858) is large, implying that minor deviations from the proposed model will be considered significant. This is a well-known limitation of the Hosmer and Lemeshow test [40].

**Table 2:** Logistic regression analyses for not described versus described patient’s experience (Step 1 in SESPI)

	Odds Ratio (95% CI)		*P* value
Education level (ref = Bachelor with further education)			
Bachelor	1.108	(.935–1.313)	.238
Other	1.049	(.852–1.293)	.650
Shift type (ref = Night)			
Day	5.308	(4.307–6.543)	<.001
Evening	6.474	(5.260–7.969)	<.001
Hospital ward (Acute vs DPC. ref. = Acute)	.822	(.700–.966)	.017

Reference category: not described experience

Of the total number of excerpts, 66.5% proceeded to Step 2 in SESPI and were assessed in terms of what kind of patient experiences were reported. The extreme score proportions of the excerpts that described patients’ experiences were as follows: – I Uncomfortable (64.9%) and + II Very positive experience (0.6%) (see Table 1). The DPC excerpts described degrees of positive experiences more often than the acute ward (+ I Positive experience: 24.0% vs. 12.5%). Moreover, 20.3% of the excerpts from the acute ward were scored in - II Very uncomfortable, whereas the percentage share for the DPC was 12.1%; t (2562) = − 8.7, *p* < .001. This difference was also significant when controlling for education level and shift type (*OR* = .442, p < .001, 95% CI = .311–.631) (see Table 3). Excerpts reported from the day and evening shifts contained more experiences in the positive spectrum than those from the night excerpts. Table 3 shows that both day and evening excerpts had about 40% decreased odds of reporting uncomfortable experiences, compared to the excerpts from the night shifts. There were no significant differences between the education categories regarding whether staff reported patients’ experiences or not (see Table 2). Neither were there revealed significant differences between the three staff groups for Step 2 (categories of recorded experiences) (see Table 3).

**Table 3:** Ordinal regression analyses for recorded patient experiences (Step 2 in SESPI)

	Odds Ratio (95% CI)		P value
Education level			0.534
Further education	1		
Bachelor	0.960	(0.782, 1.179)	0.700
Other	0.876	(0.689, 1.112)	0.277
Shift type			**0.001**
Day	0.591	(0.439, 0.794)	< 0.001
Evening	0.584	(0.437, 0.783)	< 0.001
Night	1		
Hospital ward			**< 0.001**
Acute	1		
Dpc	0.442	(0.311, 0.631)	< 0.001

### Descriptions of staff-patient interactions

A very high proportion (70.7%) of the 2564 excerpts that described patients’ experiences (*n* = 2564) did not report any approaches at all from staff (see Table 1). Almost 10% (9.3%) of the excerpts described staff approaches, but had no information on how patients had responded to these approaches. A somewhat lower proportion (8.5%) reported superficial staff approaches that patients appeared to experience as fair enough. Mostly, these approaches were oriented toward practical solutions and failed to capture patients’ experiences. Only 11.5% described both the staffs’ approaches and the patients’ responses, the two criteria that had to be met to assess attunement. According to Table 4, educational level did not explain much of the variation concerning the degree of reported staff-patient interactions. The “Other” staff group had 44% increased odds for not reporting their approach at all versus writing a complete staff-patient interaction when they were compared to the staff group with the highest level of education (*LR* = 1.444, *p* = .049, 95% CI = 1.001–2.083). This was the only significant finding for the education variable. Day and evening excerpts had almost 80% increased odds for not reporting their approaches at all compared to the night excerpts. For the category “staff approach described, but no patient’s response,” the differences compared to the night excerpts were even larger (*LR* for day excerpts = 2.898, *p* = .002, 95% CI = 1.455–5.774; *LR* for evening excerpts = 2.639, *p* = .005, 95% CI = 1.331–5.235). Because the night excerpts had lower odds for reporting patients’ experiences (which meant that many of the night reports did not proceed to Step 2 and 3 in SESPI), they were not assessed on staff-patient interactions. However, when the night excerpts did describe a patient’s experience, these excerpts had increased odds for reporting both the staff’s approach and the patient’s response compared to excerpts reported from day and evening shifts. There was only one exception to this. For the category “superficial approach,” where the staff met the patient adequately, but on a superficial level (for instance, offering anxiolytic medicine at the patient’s request when the patient was anxious), the current category *LR* for evening excerpts was .593, *p* = .048, 95% CI = .353–.996. Concerning differences between the wards regarding staff-patient interactions, excerpts from the DPC had increased odds for all the three categories presented in Table 4, showing that the acute ward had the greatest odds for reporting both their approach and the patient’s response to the actual approach. The difference in favor of the acute ward was 40–50%.

**Table 4:** Multinomial regression analyses for type of described staff-patient interactions (Step 3 in SESPI)

	Likelihood Ratio (95% CI)	*P* value
A. No staff approach (ref = D. Fully described interaction)		
Education level (ref = Bachelor with further education)		
Bachelor	1.021 (.759–1.372)	.891
Other	1.444 (1.001–2.083)	.049
Shift type (ref = Night)		
Day	1.784 (1.189–2.675)	.005
Evening	1.790 (1.201–2.667)	.004
Hospital ward (Acute vs. DPC)	1.407 (1.070–1.851)	.015
B. Staff approach, but no patient’s response (ref = D. Fully described interaction)		
Education level (ref = Bachelor with further education)		
Bachelor	1.332 (.879–2.018)	.176
Other	1.318 (.786–2.211)	.295
Shift type (ref = Night)		
Day	2.898 (1.455–5.774)	.002
Evening	2.639 (1.331–5.235)	.005
Hospital ward (Acute vs. DPC)	1.463 (1.001–2.140)	.050
C. “Superficial” staff approach (ref = D. Fully described interaction)		
Education level (ref = Bachelor with further education)		
Bachelor	1.122 (.740–1.699)	.588
Other	1.207 (.712–2.047)	.485
Shift type (ref = Night)		
Day	.619 (.365–1.048)	.074
Evening	.593 (.353–.996)	.048
Hospital ward (Acute vs. DPC)	1.519 (1.030–2.239)	.035

Only 294 out of the 3858 excerpts (7.6%) were possible to assess concerning attunement. This number of excerpts (*n* = 294) had the following score distribution: 8.2% were scored in – II Failed attunement, whereas only 0.7% were in the + II Successful attunement category (see Table 1). The most frequently reported interactions were – I Partially failed attunement, 45.2%, and + II Partially successful attunement, 45.9%. A majority of excerpts from the acute ward (62%) scored in one of the two categories of failed attunement, compared to 43% from DPC. DPC had more excerpts reporting + I Partially successful attunement; t (292) = − 4.06, *p* < .001). According to Table 5, these differences were confirmed when controlling for the other variables. There was a 72% reduced odds of reporting “on the lower part of the scale”, i.e. in the failed attunement spectrum, for the DPC excerpts, compared to the excerpts from the acute ward (*OR* = .279, *p* = .001, 95% CI = .129–.603). The evening excerpts had 64% decreased odds of reporting in the failed attunement spectrum, compared to the excerpts from the night shifts (*OR* = .360, *p* = .012, 95% CI = .163–.797).

**Table 5:** Ordinal regression analyses for recorded attunement (Step 4 in SESPI)

	Odds Ratio (95% CI)		P value
Education level			0.139
Further education	1		
Bachelor	0.543	(0.290, 1.016)	0.056
Other	0.582	(0.285, 1.188)	0.137
Shift type			**0.038**
Day	0.504	(0.224, 1.133)	0.098
Evening	0.360	(0.163, 0.797)	0.012
Night	1		
Hospital ward			**0.001**
Acute	1		
Dpc	0.279	(0.129, 0.603)	0.001

## Discussion

The aim of this study was to use SESPI to assess the quality and quantity of patients’ experiences and the documented staff approaches in response to them in a randomized sample of excerpts from nurses’ progress notes. The study showed that one third of the excerpts from the progress notes did not contain any descriptions of patients’ experiences. However, the most remarkable finding was that only 7.6% of the excerpts described how staff responded to patients’ experiences. This means that as much as 92.4% of the excerpts were impossible to assess according to attunement, which is a core feature in a therapeutic interaction [41, 42]. In a previous qualitative study [43], nurses revealed that in their training they were advised to only report observations of the patient and to avoid writing their own interpretations. The staff members considered reporting relevant information to the psychiatrist for diagnostic purposes of the highest importance, and, in this context, patients’ experiences, as well as staff-patient relationships, had limited relevance for documentation. Other researchers have questioned whether the existing record system templates restrict documentation of psychosocial and relational dimensions of the care [44, 45]. A bio medical model for inpatient psychiatry is often described as contradictory to models of care which highlights the therapeutic staff-patient relationship. However, researchers described a positive correlation between a therapeutic staff-patient relationship and adherence to medical treatment and psychotherapy [11]. Still a general understanding of reporting according to a medical model as well as limitations in the electronic documentation template could possibly explain why the vast majority of excerpts in the current study could not be evaluated in terms of whether staff approaches turned out to be therapeutic or not. Additionally, reporting policies of the wards could possibly have restricted reporting of the staff-patient interactions. This finding contrasts with what a meta study of nursing documentation highlighted as the main purpose of staff’s reports: The documentation was supposed to reflect how staff understood their patients and how they approached their patients in actual situations [17]. Patients have emphasized that staffs’ ability to understand and grasp their experiences and feelings is of paramount importance to them [46, 47]. However, the excerpts in our study rarely recorded staffs’ efforts in attuning to patients’ feelings. As far as we know, no other quantitative studies have reported quality and quantity measures for staff-patient interactions in nursing documentation in mental health contexts. Accordingly, the current study appears to represent an important first step in the effort to explore the extent and quality of documented staff-patient interactions.

Some differences between the acute and the DPC unit were found. The number of excerpts reporting patients’ experiences from the DPC 68.6% vs. acute: 64.3% was significantly larger. The DPC excerpts described patients’ experiences as less uncomfortable, and the staff-patient interactions were scored as having more successful outcomes of attunement than the interactions from the acute ward. We failed to find other studies with the same design that investigated the outcome of empathic communication. Still, it seems reasonable that patients in an acute phase of their illness may experience higher degrees of very uncomfortable emotions compared to patients in more stable phases who are admitted to an open ward like the DPC. Lorem and Hem [38] highlighted that it is challenging for healthcare workers to achieve an attuned understanding when patients are in acute phases of psychosis. Factors like, for instance, the severity of the patient’s symptoms and the staff at the acute ward having opportunities to use seclusion or mechanical restraints could be possible obstacles to attunement. This may explain why the excerpts from the acute ward more often described interactions of failed attunement. However, we also think that reporting failed attunement is important for improving the ongoing treatment process in the direction of change and with positive attunement as the main aim.

Previous research has indicated a need for evaluation and further development of therapeutic staff-patient interactions in acute mental health units [48]. Evaluation with the use of SESPI could be an important contribution to this end. For instance, in our study, excerpts from the night shift reported patients’ experiences as being more uncomfortable and described interactions as falling more in the failed attunement spectrum than reports from the evening shift did. For evaluation and improvement purposes, these failed attunement interactions could be analyzed to explore alternative staff approaches with the potential of achieving more empathic understanding and communication. Of course, progress notes can only serve as a basis for evaluation of therapeutic staff-patient interactions if they contain this type of information. In our study, the night excerpts had more descriptions of interactions than day and evening reports. It is important to note that we do not know how staff reporting actually corresponded to patients’ experiences and perceptions. Progress notes are but one source for evaluation.

The results from our study suggest that staffs’ efforts to facilitate therapeutic interactions were scarce in the nursing documentation, which is in line with findings from qualitative studies [31, 32]. Surprisingly, the study revealed no substantial differences between the staff education levels concerning reporting practices of staff-patient interactions. The small number of excerpts describing attuned interactions points to a need for reassessing staff training and nursing education regarding documentation and also to evaluate whether the documentation systems’ templates support reporting core aspects of mental health care. However, we did not conduct an observational study of the quality and quantity of attunement on psychiatric wards. Therefore, one must keep in mind that our design only allows for discussions of reporting practices regarding attunement. An increased focus on reporting interactions, in itself, could possibly create a greater consciousness of therapeutic interactions in clinical practice.

One of the current study’s strengths was the considerable number of randomized progress notes (*N* = 3858). The two mental health units chosen for this research represented a wide range of phases of patients’ illnesses, diagnoses, and degrees of coercive versus voluntary treatment. These elements are important with regard to generalizability. We used the SESPI, a previously reliability-tested instrument, for assessing staff-patient interactions for attunement [39]. Attunement is an established term related to therapeutic interactions [33, 35, 49]. In the SESPI, the categories of attunement were operationalized with examples from staff progress notes in mental health services. The results from the current study showed a spread concerning the distribution of excerpts over the four steps in SESPI (See Fig. 1). These results indicate that SESPI is applicable to measuring progress notes in terms of the quantity and quality of reported patient experiences and staff-patient interactions. However, as the current study was the first to assess staff progress notes using the SESPI and we found no comparable research with other instruments, new studies are required in different inpatient mental health contexts. The aspects of generalizability and validity concerning the SESPI instrument discussed above need to be validated against these future studies.

### Limitations

A considerable number of progress notes were randomized for this study, but only 294 described the staff-patient interactions thoroughly enough to be assessed in terms of attunement. For this reason, the results for Step 4, the percentage share for each attunement category, must be interpreted with caution.

The National Committee for Medical and Health Research Ethics in Norway (NEM) approved this study without any demand for patient’s consent on condition that the researchers had no access to personal identifying data of the patients. This approval was important to prevent systematic inclusion bias. However, the lack of information about diagnoses of the patients and psychiatric symptoms is a limitation of this study. Clearly, it is a demanding task to attune to a patient suffering from severe psychotic symptoms. Still, the cluster effects of the 90 journals used in the current research were low (<.125 in each of the four Steps of the SESPI). Therefore, future research may want to add diagnoses as an independent variable in the regression models to explore possible differences in the reporting of attunement. Additionally, in compliance with the conditions for ethical approval of this research, we had no access to staff characteristics except their educational background. Education did not explain much in our models. There was only one significant finding for this variable, and we did not find a sustainable interpretation of this result (see Table 4 for the category “other”). There could possibly be a lack of independence between the excerpts because the same staff member had written several excerpts. Due to the large number of randomized excerpts, and that about 90 persons wrote progress notes in the selection period, the risk of writer bias was low in the data set. The fact that the data did not reveal any major differences between the staff groups support this hypothesis. Nevertheless, to be on the safe side we recommend future research to control for staff background variables such as gender and years of practice in the analyses.

This is the first quantitative study evaluating nursing documentation using the SESPI. The results indicate that the SESPI has the ability to include and distinguish between different types of staff-patient interactions. However, further research is required both for validation of the results of our study and for validation of the scale. For these reasons we did not conduct analyses of possible interaction effects for the independent variables, but chose to emphasize main effect sizes in our findings. Still, in our opinion, this study is an important first step in quantitative investigation of staff-patient interactions in nursing documentation. From this perspective, the descriptive statistical findings are very important. We consider those results sustainable due to the large number of randomized excerpts. More studies on progress notes data from other mental health services, with other policies and characteristics of the patient groups, are required before the findings can be generalized to other settings. We suggest that future studies include more than the three independent variables that were available for this research. We also recommend to test possible interaction effects in regression models with larger samples.

## Conclusion

The findings from this study present a unique insight into the quality and quantity of mental health nursing documentation regarding reported experiences of patients and staff-patient interactions. Only 7.6% of the excerpts from the progress notes sufficiently described staff-patient interactions for analysis in terms of attunement. Nursing documentation is intended to reflect staffs’ care for their patients. Still, therapeutic relationships, which are considered highly relevant and important in inpatient mental health care, were almost non-existent in the documentation. These results point to a need for discussing mental health staffs documentation practices, the focus in education programs concerning documentation of mental health care, and existing electronic reporting systems’ templates in relation to core aspects in mental health care. However, as this is the first study measuring nursing documentation using the SESPI instrument, new studies are required to validate our findings.

Assessing nursing documentation regarding staff-patient interactions may generate more thorough documentation of staff-patient interactions in the future. In turn, nursing documentation may become a better source for the evaluation and development of therapeutic staff-patient interactions in clinical practice. Notably this research did not observe staff-patient interactions in practice, nor were patients’ opinions of the reported episodes sought. Both of these would be important to include in future research.

## Additional file

Additional file 1: Scale for the Evaluation of Staff-Patient Interactions (SESPI). An instrument to evaluate progress notes in nursing documentation in mental health services. (DOCX 860 kb)

## Data Availability

The datasets generated and analyzed during the current study are not publicly available due to the conditions for the ethical approval, but are available from the corresponding author on reasonable request.

## Abbreviations

DPC: An open inpatient district psychiatric centre
NEM: The National Committee for Medical and Health Research Ethics in Norway
SESPI: The Scale for the Evaluation of Staff-Patient Interactions in progress notes
